# Pregnancy Predictors after Intrauterine Insemination in Cases of Unexplained Infertility: A Prospective Study

**DOI:** 10.1155/2016/5817823

**Published:** 2016-09-21

**Authors:** Ishita Ganguly, Aparna Singh, Shilpa Bhandari, Pallavi Agrawal, Nitika Gupta

**Affiliations:** Department of Reproductive Medicine and Surgery, Sri Aurobindo Medical College and Post Graduate Institute, Indore, Madhya Pradesh, India

## Abstract

*Introduction*. Aim of the study was to find the effect of various prognostic factors in cases of unexplained infertility undergoing controlled ovarian stimulation (COS) with intrauterine insemination (IUI).* Methods*. 146 cases of unexplained infertility were included. A maximum of 3 cycles of IUI were done with clomiphene citrate/HMG. Ovulation trigger was given when the largest follicle diameter was >18 mm, and IUI was planned 36 hours later. Luteal phase support was given for 15 days, urine pregnancy test was done on day 15, ultrasonography was done at 7 weeks, and pregnancy was followed up till delivery.* Results*. A total of 146 couples have undergone 239 cycles of IUI out of which 27 had UPT positive after 15 days. 14.8% had 1st-trimester abortion while 3.7% were ectopic. 86.3% were singleton pregnancies and 13.6% were twins. CPR was 11.29% per cycle and 18.4% per couple; LBR was 9.2% per cycle. Apart from duration of stimulation (*p* = 0.037) and number of treatment cycles (*p* = 0.045), no other factors had significant prognostic value.* Conclusion*. For unexplained infertility, IUI can be done to provide patients with the time that they need before moving on to IVF while providing a respectable chance of pregnancy.

## 1. Introduction

Unexplained infertility is a vexing state for the subfertile couple as the clinician is often unable to either provide definitive and demonstrable cause of infertility or provide a concrete line of management for the same. Role of ovulation induction with planned intercourse is controversial in already ovulating patients. Controlled ovarian stimulation (COS) with homologous intrauterine insemination (IUI) or in vitro fertilization (IVF) became some of the treatment options available. In most cases absence of fertilization or implantation has been held responsible for the absence of pregnancy and consequentially IVF is deemed as a logical choice of treatment. This approach is often felt to be too aggressive in countries like India where in vitro fertilization is yet to find universal acceptance. IVF is also a costlier treatment which may not be an option at all for a significant percentage of Indian subfertile population; ready availability of IVF facility also is a big hindrance for providing this option to cases in whom it is indicated. In view of the above factors, in a significant number of cases IUI is often the most practical treatment choice for the treating physician as well as the couple before moving on to IVF. IUI is a relatively less expensive and less invasive procedure; it is based on the principle of “increasing the number of gametes (sperms and oocytes) at the right place at the right time.” Success rate of controlled ovarian stimulation (COS) with intrauterine insemination (IUI) varies between 8 and 22% [1–3]. Various prognostic factors like age of the couple, duration of infertility, BMI, semen parameters, duration of stimulation, and endometrial thickness have been studied in the past to find their effect on success rate. However most of the studies included all the indications for IUI like mild male factor, endometriosis, anovulation, and cervical factors. The following study was conducted to find the prognostic factors in cases of unexplained infertility undergoing COS with IUI.

## 2. Material and Methods

A prospective study was conducted in the Department of Reproductive Medicine at a tertiary-care centre between Jan 2014 and Dec 2015. Patients of unexplained infertility undergoing COS with IUI were included in the study. Approval for the study was taken from the ethics committee.

Unexplained infertility was defined as cases where the basic infertility workup (ovulatory cycles, normal uterine cavity, at least one patent tube on hysterosalpingography {HSG} or laparoscopy, and normal semen parameters according to WHO 2010 criteria) was found to be normal.

Patients included in the study were cases of unexplained infertility of the age group 20–40 years, with total antral follicle count >10, have not undergone IUI in the past, and had ≤3 follicles on the day of ovulation trigger.

Patients with uterine pathology diagnosed on TVS like fibroid uterus, adenomyosis, or endometrioma were excluded from the study.

A maximum of 3 cycles of IUI were done. Controlled ovarian stimulation was done using clomiphene citrate 100 mg from day 2 of menses (after confirming absence of ovarian cyst and endometrial thickness <5 mm) for 5 days with gonadotropin HMG (Materna HMG, Emcure) 75 IU every alternate day starting from day 5 till the day of ovulation trigger. Follicular monitoring was started from day 7 based on which the day of ovulation trigger was determined. Ovulation trigger was planned when the largest follicle diameter was >18 mm using injection hCG (Materna hCG, Emcure) 5000 IU intramuscularly and IUI was planned 36 hours later. Husband was instructed to give the semen sample by masturbation in a sterile wide mouth container with abstinence of 2–7 days.

Semen preparation was done using a density gradient method. The semen sample was allowed to liquefy and then layered over 80/40 (Pureception, Sage IVF, Trumbull, USA) density gradient in a ratio of 1 : 1 and centrifuged at 2000 rpm for 10 min. The supernatant was discarded and pellet was mixed with 0.5 mL SPM; it was then mixed with 2.5 mL SPM in another conical tube and centrifuged at 1500 rpm for 5 minutes. The supernatant was discarded and the pellet was again layered with 0.5 mL of SPM and sperms were allowed to swim up at 37 degrees Celsius for 15 minutes. 0.5 mL supernatant is loaded in a soft IUI catheter. Pre- and postwash semen analysis was done using WHO 2010 guidelines. Patients with semen parameters previously normal but found to be abnormal on the day of IUI were not excluded from the study.

IUI was done under transabdominal ultrasonography guidance with full bladder using Wallace soft IUI catheter. Patient was asked to lie down in slight head low position for 30 minutes. Luteal phase support was in the form of micronized progesterone vaginal suppository 200 mg twice daily for 15 days. Serum beta hCG was done on day 15 to calculate the pregnancy rate. Values above 100 mIU/mL were considered positive. Ultrasonography was done at 7 weeks to determine the clinical pregnancy rate (CPR) and followed up till delivery to calculate the live birth rate (LBR).

Age, marriage duration, days of stimulation, number of dominant follicles more than 14 mm in diameter, endometrial thickness, number of cycles, body mass index (BMI), pregnancy rate, CPR, LBR, total motile fraction, and % of normal sperm morphology were noted. All the patients who had a positive pregnancy test on day 15 were considered in the “positive” group while patients who had a negative pregnancy test on day 15 were considered in the “negative” group and these two groups were compared.

Student's *t*-test was applied to difference of mean of quantitative variables. Chi-square test was applied to study the difference of frequency.

## 3. Result

A total of 146 couples have undergone 239 cycles of IUI out of which 27 had UPT positive after 15 days. Four (14.8%) had 1st-trimester abortion while one (3.7%) was ectopic and had to undergo laparoscopic salpingectomy. 19 (86.3%) were singleton pregnancies and three (13.6%) were twins. CPR was 11.29% per cycle and 18.4% per couple; LBR was 9.2% per cycle. On basis of the result of serum beta hCG the cycles were divided in two groups: positive and negative.

Demographic distribution like age, BMI, duration of infertility, type of infertility (primary or secondary) was the same among both the groups (positive and negative). 172 were of primary infertility while 67 were of secondary infertility. There was no significant difference in the pregnancy rate in cases of primary (10.46%) and secondary infertility (13.43%) group (*p* value 0.503) (Table 1).

There was a decreasing trend in pregnancy rate with increasing age from 13.7% in <25 years' age to 10.22% in age group of 30–34 years but it slightly increased in the age group of >35 years though the difference was not significant (*p* value 0.93).

There were 146 first-treatment cycles, 68 second-treatment cycles, and 25 third-treatment cycles. Clinical pregnancy rate was 15.75%  and 5.88% per cycle during the first and second cycle, respectively, while none conceived during the third cycle. This difference was significant with a *p* value of 0.045. So among the conceived patients 85.19% conceived during the first cycle while only 14.81% conceived during the 2nd cycle and none during the 3rd cycle.

Number of dominant follicles (*p* = 0.077) and endometrial thickness (*p* = 0.748) on the day of trigger were similar in both the groups. There were 7 patients who had ET < 5 mm; none of them conceived, but the finding was not significant. However the duration of stimulation was significantly longer in the conceived group (12.92 ± 2.99) compared to the nonconceived group (11.39 ± 2.05) with a *p* value of 0.037.

Semen parameters like total motile fraction and morphology were similar in both the groups (*p* value 0.05 and 0.403, resp.).

## 4. Discussion

According to the practice committee of American Society for Reproductive Medicine, guidelines have been published for basic infertility workup [4]. They have included ovulation assessment, hysterosalpingogram, husband semen analysis, uterine cavity assessment, and, if indicated, tests for ovarian reserve and laparoscopy [4]. Unexplained infertility is thus a diagnosis of exclusion when the basic infertility workup is found to be normal. The treatment of unexplained infertility is often empiric as there is no specific treatment for a specific defect or functional impairment. The various modalities of treatment available are expectant treatment (planned intercourse with lifestyle changes), ovarian stimulation with either clomiphene citrate (CC) or CC with gonadotropins or only gonadotropins followed by intrauterine insemination, and in vitro fertilization. Ovulation stimulation without IUI has been discouraged lately because a combined analysis of the evidence showed that 40 cycles of ovulation induction without IUI were required to achieve 1 extra pregnancy [4]. The theoretical reason behind ovarian stimulation in an already ovulating patient is to overcome the subtle ovulatory defects which cannot be diagnosed due to limitation of the available tests; at the same time increasing the number of follicles will increase the chances of fertilization and subsequent pregnancy rate. Intrauterine insemination involves introduction of prepared semen sample into the uterine cavity directly thus bypassing any undiagnosed cervical factor and at the same time increasing the concentration of motile sperms near the actual site of fertilization, that is, the fallopian tube. Thus the combined approach of ovarian stimulation with IUI has been found to be helpful. The ASRM practice committee has published an analysis of the previously available data to study the cost effectiveness of the various treatment options for patients with unexplained infertility [4]. The analysis showed that as the pregnancy rate/cycle increases so does the treatment cost. IVF has been found to be associated with a higher live birth but due to financial, social, or personal reasons patient might opt for a less expensive and less invasive option. A Cochrane review by Pandian et al. has mentioned that IVF has higher live birth compared to expectant management, unstimulated IUI, and IUI + gonadotropins (pretreated with clomiphene + IUI) but in treatment-naïve patients there is no conclusive evidence of difference in live birth between IVF and IUI + gonadotropins/clomiphene [5]. Based on the couples factors like age, duration of infertility, ovarian reserve, and previous treatment history, the treatment plan needs to be individualised. So for unexplained infertility IUI can be done to provide patient with the time that they need before moving on to IVF while providing a respectable chance of pregnancy. The number of cycles that should be advised before moving on to IVF has been a matter of debate as the cumulative pregnancy rate increases with the number of IUI attempts. There is evidence suggesting that the number of IUI trials should be limited to 3 as the pregnancy rate per cycle is very low after the 3rd cycle [6].

There is another subject of debate regarding single versus double IUI; several studies have been done but most of them included all causes of infertility. Some of the studies have found improvement in pregnancy rate but most of the randomised trials have denied any benefit as there is no statistical significance [7]; therefore in the present study we have done single IUI per cycle.

The objective of this study was to study the impact of various prognostic factors on the pregnancy rate in cases of unexplained infertility, so that it can help in counselling patients as well as deciding the appropriate treatment option available based on patient factors.

In the present study pregnancy rate per cycle was 11%. Isa et al. found pregnancy rate of 8.45% in cases of unexplained infertility. Ashrafi et al. found pregnancy rate per cycle as 19.9%; best results were found in patients of unexplained, primary infertility, less than 5-year duration, and IMC (inseminated motile sperm count) > 30 × 10^6^ [8, 9].

Age of the couple, especially female age, has been found to be an important predictor in many studies like Montanaro Gauci et al. in 2001 and AMIGOS trial in 2016 [10, 11]. Based on these studies it was believed that advancing age adversely affects oocyte number, oocyte quality, corpus luteal function, and endometrium and thus decreases the pregnancy rate. However study by Isa et al. in 2014 [8] found no association of pregnancy rate with age similar to our study. The possible explanation could be that the patients were <40 years in our study and that ovarian stimulation improves the follicle and endometrial development and the resultant good quality corpus luteum prevents luteal phase defect.

Duration of infertility is another prognostic factor studied with conflicting findings in different studies. Hansen et al., Kamath et al., Tomlinson et al., and Ashrafi et al. in their independent studies found prolonged duration of infertility to be associated with decreased success rate. Similar to the present study, Zainul et al. and Tay et al. did not find any significance associated with duration of infertility [9, 11–15].

Multifollicular growth has been found to be associated with improved chances of pregnancy in studies by Nuojua-Huttunen et al., Ibérico et al., and Dickey et al. [2, 16, 17]. But multifollicular growth is associated with risk of multiple pregnancy so cycle is cancelled if >3 follicles are dominant (>14 mm). In the present study the number of dominant follicles/cycles (2.14 ± 1.14 versus 1.91 ± 0.96) was more among the patients who conceived but the difference was not significant. 13.6% of pregnancies were twins.

Body mass index has also been studied as a prognostic factor. Obesity has been found to be associated with anovulatory infertility due to the changes in sensitivity to insulin and androgen which affects hormonal milieu. In study by Wang et al. and Dodson and Haney no association with BMI was found which is similar to our study (*p* value 0.08). The possible reason could be that our study cases were not anovulatory [18, 19].

Endometrial thickness was found to be slightly higher in those who conceived (0.8 versus 0.7; *p* value 0.748) but the difference was not significant. Similar findings were found by previous studies [20–22]; however others have found endometrial thickness to be a significant factor [23–25]. 87 patients had endometrial thickness less than 7 on day of trigger of which 6 conceived (pregnancy rate 6.8%). Among those who conceived in the thin ET group (<7 mm) the average duration of stimulation was 13 days while those who did not conceive were stimulated for an average of 10.8 days, so maybe as the duration of stimulation increased, the negative effect of clomiphene on the endometrium reduced.

Among the male factors total motile fraction and morphology were studied but no significant difference (*p* value of 0.05 and 0.403, resp.) was found similar to study by Nuojua-Huttunen et al. The possible reason could be that in the unexplained infertility group males were normozoospermic [2].

Number of IUI cycles has been found to be significant with a *p* value of 0.045; most of the patients conceived during the 1st cycle while the remaining conceived in the 2nd cycle and none conceived during the 3rd cycle (Table 1). The principal weakness of this study is the small sample size and high dropout rate as very few patients were followed up till the third cycle. A high dropout rate could be due the possible change of plan as the patient is dissatisfied with the treatment and frustrated with repeated hospital visits for injection and follicular monitoring. Land et al. [26] studied the reasons for dropout in IVF program at a centre where treatment was free for the first three cycles.

Dropout rate was 26%, 33%,  and 66% after the 1st, 2nd, and 3rd cycle, respectively. It was seen that dropout after 1st two cycles was due to poor prognosis while after the third cycle it was due to financial reasons.

Duration of stimulation was found to be significantly associated with success (*p* = 0.001). 12.92 ± 2.99 days was the average duration of stimulation among those who conceived; that is, when ovulation occurred around the time of natural cycle the follicle growth was optimum and probably the endometrium was in-phase with the developing embryo with better receptivity and hence better pregnancy rate.

In our study, a trend toward reduction in success rate with increasing female age was noted (Table 2), though the success rate was slightly better in >35 years' age group compared to 30–35 years; it could be due to the small sample size in this group (*n* − 15) that the difference was not statistically significant. However, many studies have documented a significant drop in the success rate beyond the age of 40 years, with reported live births being as low as 1.4% [6, 15, 16].

It would be helpful for the couples and clinicians if a prediction model for IUI could be devised. One such prediction model for pregnancy after IUI has been validated externally by Leushuis et al., but it still lacks the impact analysis; it also has poor discrimination (AUC 0.59) [27]. If a prediction model could be developed in future, which is accurate and precise, it would help to develop guidelines regarding course of infertility treatment based on various factors of the couple.

The limitation of this study was that LH surge was not calculated to schedule the timing of ovulation trigger.

## 5. Conclusion

Treatment of unexplained infertility without a known cause is often difficult. COS with IUI and IVF offers better chances of success compared to expectant management. In our study various factors for COS/IUI were studied of which duration of stimulation and number of treatment cycles were found to significantly predict the success. The overall pregnancy rate per cycle in our study was 11.29% while the live birth rate was 9.2% and 86.3% were singleton pregnancies. Most of the other variables did not prove to have any significance. With a low success rate seen in COS with IUI in cases of unexplained infertility, IVF appears to be a logical treatment of choice especially for patients coming from long distance to a tertiary-care centre where repeated hospital visits for multiple IUI cycles might be not possible. A well formulated prediction model would help in decision making for both the treating physician and couple based on the factors present.

## Figures and Tables

**Table 1:** Descriptive variables for 239 IUI cycles.

Parameters	Positive	Negative	*p* value

Age (years)	28.15 ± 4.93	28.20 ± 4.22	0.951
Husband age (years)	32.74 ± 5.9	32.55 ± 4.83	0.856
BMI (kg/m^2^)	23.62 ± 3.46	23.42 ± 4.49	0.82
Duration of infertility (years)	6.09 ± 3.91	6.12 ± 3.68	0.971
Duration of stimulation (days)	12.92 ± 2.99	11.43 ± 2.05	0.001^*∗*^
Endometrial thickness (cm)	0.8 ± 0.16	0.75 ± 0.18	0.136
Number of follicles/cycles	2.14 ± 1.14	1.91 ± 0.96	0.077
Semen: total motile fraction	10.38 ± 5.44	8.35 ± 4.98	0.05
Semen: normal morphology (%)	6.07 ± 1.17	5.8 ± 1.6	0.403
Primary infertility	18 (10.46)	154	0.503
Secondary infertility	9 (13.43)	58	
Cycle number			
1	23	123	0.045^*∗*^
2	4	64	
3	0	25	

^*∗*^ *p* value significant.

**Table 2:** Age-wise description of IUI outcomes.

Age (years)	Positive	Negative	Pregnancy rate	*p* value

<25	7	44	13.7%	0.936
25–29	9	74	10.84%	
30–34	9	79	10.22%	
≥35	2	15	11.76%	
